# Mifepristone Derivative FZU-00,003 Suppresses Triple-negative Breast Cancer Cell Growth partially via miR-153-KLF5 axis

**DOI:** 10.7150/ijbs.39491

**Published:** 2020-01-01

**Authors:** Rong Liu, Haijun Chen, Ping Zhao, Chuan-Huizi Chen, Huichun Liang, Chuanyu Yang, Zhongmei Zhou, Xu Zhi, Suling Liu, Ceshi Chen

**Affiliations:** 1 Key Laboratory of Animal Models and Human Disease Mechanisms of the Chinese Academy of Sciences and Yunnan Province, Chinese Academy of Sciences, Kunming Institute of Zoology, Kunming, Yunnan 650223, China;; 2 Center for Excellence in Animal Evolution and Genetics, Chinese Academy of Sciences, Kunming, PR China;; 3 College of Chemistry, Fuzhou University, Fuzhou, Fujian 350108, China;; 4 Department of Breast Surgery, Yunnan Cancer Hospital, The Third Affiliated Hospital of Kunming Medical University, Kunming, Yunnan, 650118, China;; 5 Center for Reproductive Medicine, Department of Obstetrics and Gynecology, Peking University Third Hospital, Beijing 100191, China;; 6 Key Laboratory of Breast Cancer in Shanghai, Cancer Institute, Department of Breast Surgery, Fudan University Shanghai Cancer Center, Shanghai 200032, China;; 7 Institutes of Biomedical Sciences, Fudan University, Shanghai 200032, China;; 8 KIZ-CUHK Joint Laboratory of Bioresources and Molecular Research in Common Diseases, Kunming Institute of Zoology, Chinese Academy of Sciences, Kunming, 650223, China.

**Keywords:** FZU-00, 003, KLF5, breast cancer, MIF derivatives

## Abstract

Triple-negative breast cancer (TNBC) is one of the most malignant breast cancers lacking targeted therapeutics currently. We recently reported that mifepristone (MIF), a drug regularly used for abortion, suppresses TNBC cell growth by inhibiting KLF5 expression via inducing miR-153. However, its anticancer efficacy is only modest at high dose. In order to enhance the anticancer activities, a focused compound library containing 17 compounds by altering the sensitive metabolic region of mifepristone has been designed and synthesized. We first tested the cell growth inhibitory effects of these compounds in TNBC cell lines. Among them, FZU-00,003 displayed the most potent efficiency. FZU-00,003 suppresses TNBC cell growth, cell cycle progression and induces apoptosis more effectively than MIF does. Consistently, FZU-00,003 induces miR-153 expression and suppressed KLF5 expression at much lower dosages than MIF does. Furthermore, FZU-00,003 inhibits tumor growth more potently than MIF does. Taken together, the MIF derivative, FZU-00,003 may serve as a better therapeutic compound for TNBC than MIF.

## Introduction

Classically, breast cancer can be divided into three subtypes, including estrogen receptor (ERα) positive w/o progesterone receptor (PR) positive luminal subtype, human epidermal growth factor receptor 2 (HER2) positive HER2 subtype, and ER-/PR-/HER2- triple-negative subtype (TNBC). Among these subtypes, TNBC constitutes 10%-20% of all breast cancers, and is usually of higher grade and biologically more aggressive 1. TNBC has been challenging due to the heterogeneity of the disease, the absence of well-defined molecular targets and effective targeted therapies 2. Therefore, the adjuvant chemotherapy is the only choice. Even so, the prognosis of TNBC is not optimistic, more than 70% of women with metastatic TNBC die within 5 years 3. Thus, it is urgently needed to discover new targets and develop mechanism-based, effective targeted therapies for this aggressive type of breast cancer 4. Although efforts have been made to achieve this goal by numerous investigations, such as PARP1 inhibitors 5, EGFR inhibitors 6, mTOR inhibitors 7, and bromodomain inhibitors 8, 9 have been reported to display anticancer efficacy in TNBC models. Despite some initial success, most of the agents have shown only limited efficacy in both preclinical and clinical settings. Therefore, only a few of them are advanced into clinical trials for the treatment of TNBC patients 10.

Human Krüppel-like factor 5 (KLF5) has been implicated in promoting breast cell proliferation, survival, stemness and tumorigenesis 11-14. Data from clinical samples indicated that KLF5 is highly expressed in basal-type breast tumors 15, and high levels of KLF5 are positively correlated with shorter survival for breast cancer patients 16, 17. We previously identified that KLF5 is specifically highly expressed in basal TNBC cell lines 18, and depletion of KLF5 significantly suppresses basal TNBC cell proliferation, survival and tumor growth 19, 20. Our recent work implicated that pharmacological inhibition of KLF5 could suppress TNBC cell growth 13, 14. These data implicated that KLF5 could serve as a promising target for TNBC.

Recently, we found mifepristone (MIF), a synthetic progesterone receptor (PR) antagonist, which has been widely used as an abortifacient and emergency contraceptive for decades 21, suppresses TNBC cell proliferation and tumor growth partially through inhibiting KLF5 expression via inducing miR-153 13. Although MIF shows anti-TNBC activity, the potency is relatively moderate (with a half maximal inhibitory concentration (IC_50_) of 10-22 µM for TNBC cells), which has limited its further clinical application for TNBC treatment 13.

To develop more potent anti-TNBC reagents, we designed and synthesized 17 MIF derivatives, and compared the anticancer efficacy of MIF and its derivatives. We found several analogs suppressed TNBC cell viability more potently than MIF did, and FZU-00,003 had the lowest IC_50_ when compared to other derivatives. Furthermore, we demonstrated that FZU-00,003 suppressed TNBC cell growth, cell cycle progression and induced apoptosis more effectively than MIF did. FZU-00,003 induced miR-153 expression and suppressed KLF5 expression at much lower dosages than MIF does. Finally, we evaluated the anticancer efficacy of FZU-00,003 in a patient-derived xenograft model and found FZU-00,003 inhibited tumor growth more potently than MIF did. Taken together, our data suggest that the MIF derivative, FZU-00,003 may serve as a better lead compound for TNBC than MIF.

## Materials and methods

### Cell Culture and Transfection

All cell lines were purchased from the American Type Culture Collection (ATCC) and were validated by the STR analysis (Kunming Cell Bank, Kunming Institute of Zoology, Chinese Academy of Sciences). HCC1937 and HCC1806 were cultured in RPMI-1640 medium (HyClone, Logan, UT) supplemented with 5% fetal bovine serum (FBS). SUM149PT was cultured with Ham's F-12 medium (HyClone, Logan, UT) supplemented with 5% FBS, 0.5 µg/ml hydrocortisone and 10 µg/ml insulin. T47D and BT474 cell lines were maintained with RPMI-1640 medium supplemented with 5% FBS and 10 µg/ml insulin. MCF7 and SKBR3 cells were cultured in DMEM medium with 5% FBS. All cells were maintained at 37 ^o^C with 5% CO_2_ in a humidified atmosphere.

MicroRNA inhibitor (Ribobio, Guangzhou, China) was transfected into different cell lines using Lipofectamine 2000 following the manufacturer's manual (Invitrogen, Carlsbad, CA, USA).

### Cell Viability Assays

Cell viability was quantified with the sulforhodamine B (SRB) assay as described previously 13, 22. Briefly, 1-4 × 10^4^ cells per well were plated in 48-well plates and treated with mifepristone, mifepristone derivatives or DMSO at designed concentrations for 48 hours. The cells were then fixed with 10% trichloroacetic acid at 4 °C overnight followed by washing with deionized water 5 times. The cells were stained with 0.4% (W/V) SRB in 1% acetic acid for 15 min at room temperature. The plates were washed 5 times with 1% acetic acid and dried. Finally, 10 mM Tris base was added to dissolve the dye and the optical densities at 530 nm were determined at a spectrophotometric plate reader (Epoch, Bio-Tek, USA).

### Cell Proliferation Assay

Cell proliferation of SUM149PT and HCC1937 cells was measured using Click-iT EdU Alexa Fluor 488 Imaging Kit (Invitrogen) following the manufacturer's instructions. Briefly, drug treated cells were incubated with EdU solution for 4 hours before being fixed using 3.7% formaldehyde. After washing with 3% BSA (bovine serum albumin), cells were permeabilized by 0.5% Triton X-100 (Sigma) and incorporated EdU were detected by Alexa Fluor azide. Nuclei were stained with Hoechst 33342. The total numbers of cells and EdU-incorporated cells in each sample were counted using a fluorescent microscope.

### Cell Cycle Analysis

Drug or DMSO treated cells were trypsinized, harvested, washed once with 1 × PBS and fixed with 70% ethanol at 4 °C overnight. Fixed cells were washed with 1 × PBS and stained with propidium iodide (PI) buffer (0.05mg/ml PI, 0.3% NP-40, 0.5mg/ml RNase A) for 30 min in dark at room temperature and subjected to cell cycle analysis via Accuri™ C6 flow cytometry (BD bioscience, San Diego, CA) within 4 hours.

### Apoptosis Analysis

Trypsinized cells were collected and washed by 1×PBS once. Cells were counted and 1× 10^5^ cells stained with anti-Annexin V antibody and propidium iodide following the manufacturer's protocol (eBioscience, San Diego, CA). Briefly, the cells were washed with 1×binding buffer followed by staining with FITC-labeled annexin V in dark for 30 min at RT. Following annexin V staining, the cells were washed with 1×binding buffer twice, stained with propidium iodide, and analyzed on the Accuri C6 flow cytometer (BD bioscience).

### Western Blot Analysis and Antibodies

Cell lysates were prepared as described previously 23. Briefly, cells were collected in cell lysis buffer for protein extraction, and 40 μg protein samples were subjected to SDS-PAGE and blotted onto polyvinylidene fluoride (PVDF) membranes. Membranes were blocked with 5% non-fat milk for 1 hour at RT and then incubated with the specific primary antibodies at 4°C overnight. After washing with PBS containing 0.1% Tween-20 (PBST), the membranes were incubated with horseradish peroxidase (HRP) conjugated secondary antibodies (Jackson ImmunoResearch Laboratory, West Grove, PA) for 1 hour at RT. The Membranes were washed with PBST and incubated with Western Lighting Chemiluminescence Reagent Plus (PerkinElmer Life Sciences, Shelton, CT) and then the targeted proteins were visualized on an ImageQuant LAS4000 Biomolecular imager (GE, PA).

The anti-PARP and p21 antibodies were purchased from Cell Signaling Technology (Danvers, MA). The anti-p27 antibody was from Becton Dickinson (San Diego, CA).The anti-KLF5 rabbit polyclonal antibody has been described previously 23.The anti-β-actin antibody was from Sigma (St. Louis, MO).

### microRNA Reverse-transcription and qPCR

HCC1937 and SUM149PT cells were treated with FZU-00,003, MIF or DMSO control at indicated dosages for 24 hours. Total mRNAs were isolated using TRIzol^®^ reagent (Invitrogen). Reverse transcription was performed using the TaqMan^®^ MicroRNA Reverse Transcription Kit (Thermo Fisher Scientific, Fremont, CA) and miRNA levels were quantified using RT Real-Time^TM^ SYBR Green/Rox PCR master mix (SAbiosciences, CA) on the ABI-7900 system. Primers for U6 and miR-153 are listed in supplementary table 1.

### Stable Over-expression of KLF5

The KLF5 cDNA was amplified and cloned into pCDH-puro lentiviral vector using forward primer, 5'-AGAGAATTCGGATCCATGGCTACAAGGGTGCTG-3' and reverse primer 5'-CTTCCATGGCTCGAGTCAGTTCTGGTGCCTCTTC-3'. To prepare the lentivirus, all lentiviral plasmids and the packing plasmids were co-transfected into HEK293T cells using Lipofectamine 2000. Lentiviruses were collected at 72 hours after transfection and used to transduce HCC1937 cells in 6-well plates. 24 hours after transduction, puromycin (1 µg/ml) was added to select drug-resistant cell populations.

### Tumorigenesis in BALB/c Nude Mice

This study was approved by the institutional ethics committees of Kunming Institute of Zoology, Chinese Academy of Sciences. Eighteen 6-week-old female BALB/c nude mice were purchased from SJA Laboratory Animal Co., Ltd. (Hunan, China). The ERα-PR-HER2- invasive ductal carcinoma UM1 patient-derived xenograft (PDX) tissues, which were maintained in fat-pads of BALB/c nude mice without culture *in vitro*, were collected and dissociated as described previously 13. UM1 cell suspension (1×10^6^ cells/point) was implanted into mammary fat pads of the BALB/c nude mice. Tumor size was measured using Vernier calipers once tumors became palpable. Tumor volumes were calculated using the following equation: tumor volume (cm^3^) = π × (length × width^2^)/6. When the tumor size reached 50 mm^3^, mice were randomly and equally distributed into three groups, which were treated with the vehicle control, 1 mg of FZU-00,003 or 1 mg of mifepristone via daily intraperitoneal injections. Tumor size was monitored twice/week for 4 weeks. All mice were sacrificed at the end of the experiment and tumors were harvested for analysis.

### Statistical Analysis

The cell cycle assay, cell apoptosis analysis and the cell viability assay were conducted in triplicate. When appropriate, the data were pooled to generate means ± standard deviation and were analyzed by t-test. P-values less than 0.05 were considered to be significant.

## Results

### Several MIF derivatives decreases TNBC cell viability more potently than MIF

**Figure 1:**
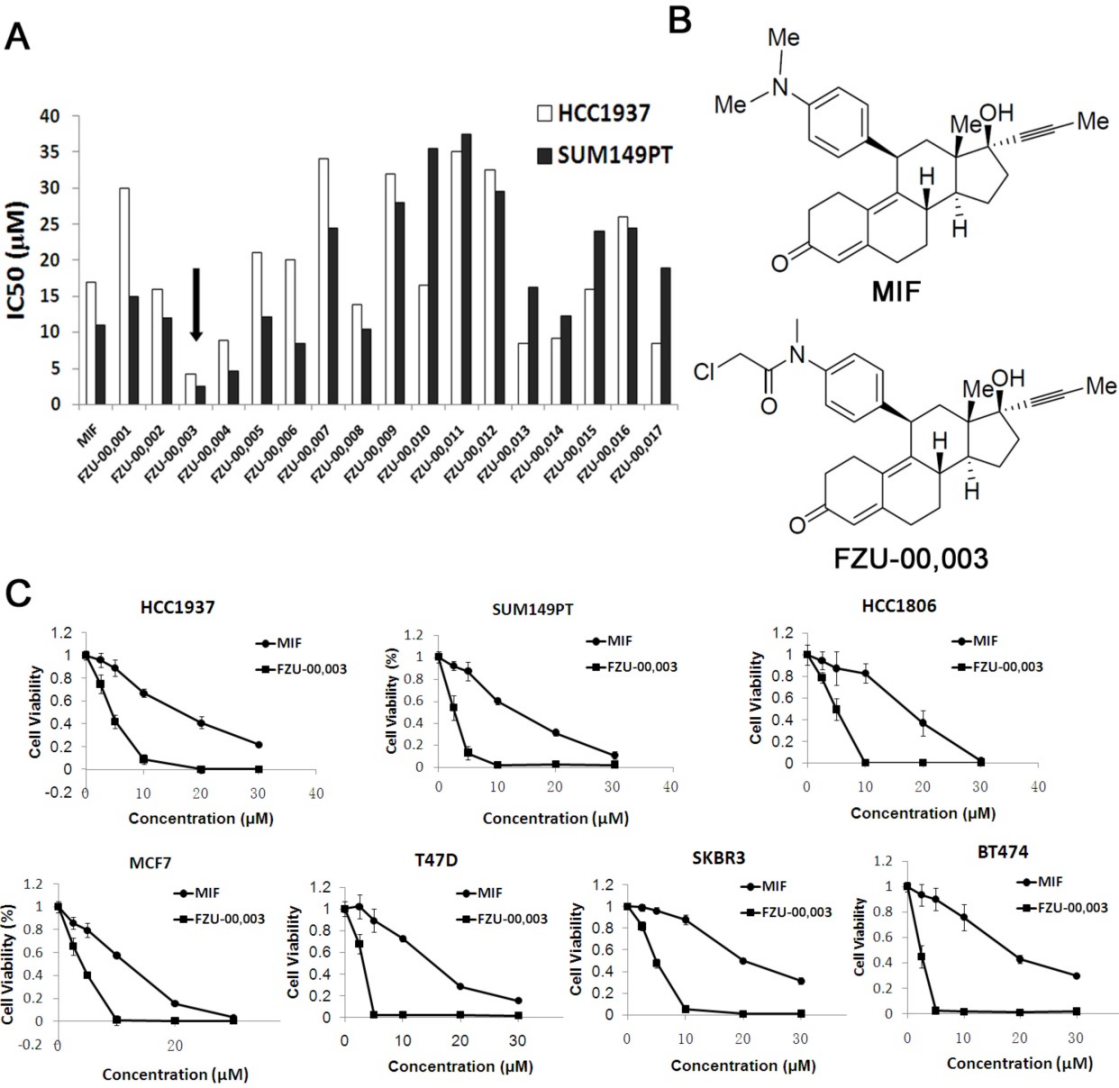
** FZU-00,003 was identified as a more potent anti-cancer compound derived from mifepristone in breast cancer cells. A.** Effects of new Mifepristone derivatives on cell survival of HCC1937 and SUM149PT TNBC cell lines. **B.** Chemical structures of MIF and FZU-00,003. **C.** The cytotoxicity of FZU-00,003 and MIF in 7 different breast cancer lines. The cells were treated with compounds at indicated dosages for 48 hours, and cell viability was measured using the SRB assay.

In order to develop more potent MIF based anti-cancer reagents, we synthesized 17 analogs based on the chemical structure of mifepristone by altering the sensitive metabolic region (Supplementary Table 2). Our preliminary screening results (Fig. 1A) revealed that N-monodemethyl mifepristone (FZU-00,001) without methyl moiety is not favorable for potency. Therefore, we directed our chemical optimization effort on this region via simple amide synthesis. We first investigated the growth inhibitory potency of these newly synthesized mifepristone derivatives in TNBC cell lines SUM149PT and HCC1937 using SRB assays. As the data shown in figure 1A, several new analogs, such as FZU-00,003 and FZU-00,004, displayed enhanced anticancer activities and suppressed cell viability more potently than MIF. Among all these derivatives, FZU-00,003 (Fig. 1B), had the most potent efficiency of inhibiting TNBC cell survival. Compared to MIF, the IC_50_ values of FZU-00,003 reduced from 17.2 μM to 4.3 μM and 11.3 μM to 2.6 μM for HCC1937 and SUM149PT, respectively.

In order to figure out whether FZU-00,003 could suppress survival of breast cancer cells other than HCC1937 and SUM149PT cell lines, we also treated 5 other breast cancer cell lines (including another TNBC cell line HCC1806, two luminal breast cancer cell lines MCF7 and T47D, and two HER2+ cell lines SKBR3 and BT474) with FZU-00,003. MIF was also tested for comparison. As shown in figure 1C, FZU-00,003 reduced cell viability at much lower concentrations than MIF in all cell lines tested. Overall, FZU-00,003 reduced breast cancer cell viability much more effectively than MIF did.

### FZU-00,003 suppresses TNBC cell proliferation more potently than MIF

**Figure 2:**
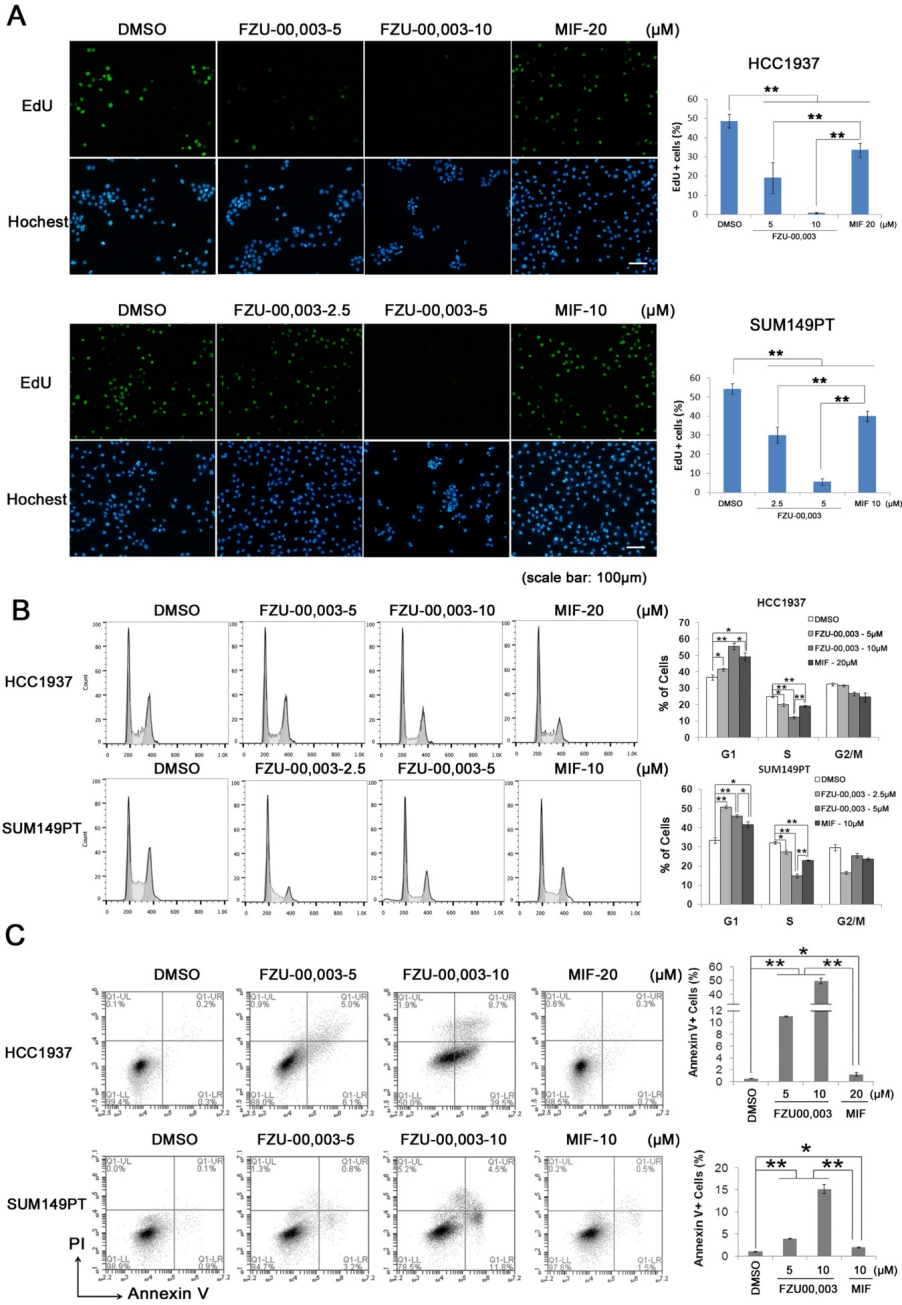
** FZU-00,003 suppresses TNBC cell proliferation and promotes apoptosis. A.** FZU-00,003 suppressed HCC1937 and SUM149PT cell proliferation more efficiently than MIF in a dosage-dependent manner. DNA synthesis of FZU-00,003 or MIF treated cells were examined using the Click-iT^TM^ EdU Alexa Fluor^®^ 488 Imaging Kit. The quantitative results are shown on the right. **B.** FZU-00,003 inhibited HCC1937 and SUM149PT G1/S cell cycle progression more potently than MIF did. HCC1937 and SUM149PT cells were treated with FZU-00,003 or MIF for 24 hours. The cells were then collected and fixed for cell cycle analysis. **C.** FZU-00,003 induced more apoptosis in HCC1937 and SUM149PT than MIF did. HCC1937 and SUM149PT cells were treated with FZU-00,003 or MIF at indicated concentrations for 24 hours. The cells were then collected for Annexin V staining and flow cytometry analysis. *, P<0.05, **, P<0.01, t-test.

It is well known that both cell growth inhibition and cell death result in reduction of cell viability. To determine the mechanism by which FZU-00,003 reduced cell viability better than MIF, we first tested whether FZU-00,003 inhibits cell growth through affecting cell proliferation. The EdU-incorporation assay was performed in HCC1937 and SUM149PT to detect the TNBC cell proliferation. As shown in figure 2A, the percentages of EdU-positive cells decreased in dosage-dependent manners in both TNBC cell lines when the cells were treated with FZU-00,003 and MIF. FZU-00,003 inhibited DNA synthesis in both cancer cell lines at much lower concentrations than MIF did. To confirm this result, we examined cell cycle progression. Consistently, FZU-00,003 inhibited cell cycle progression, especially G1/S progression, in a dosage-dependent manner (Fig. 2B). As expected, FZU-00,003 suppressed G1/S cell cycle progression at much low dosages compared to MIF.

Additionally, FZU-00,003 induced apoptosis was analyzed by the Annexin V staining. Flow cytometry analysis showed that FZU-00,003 induced apoptosis in both HCC1937 and SUM149PT cell lines in a dosage-dependent manner (Fig. 2C). FZU-00,003 significantly induced apoptosis at a concentration of 5 μM while MIF only induced moderate apoptosis at 10-20 μM. These results implicated that the anticancer activity of FZU-00,003 is stronger than MIF.

### FZU-00,003 suppresses KLF5 expression more potently than MIF

**Figure 3:**
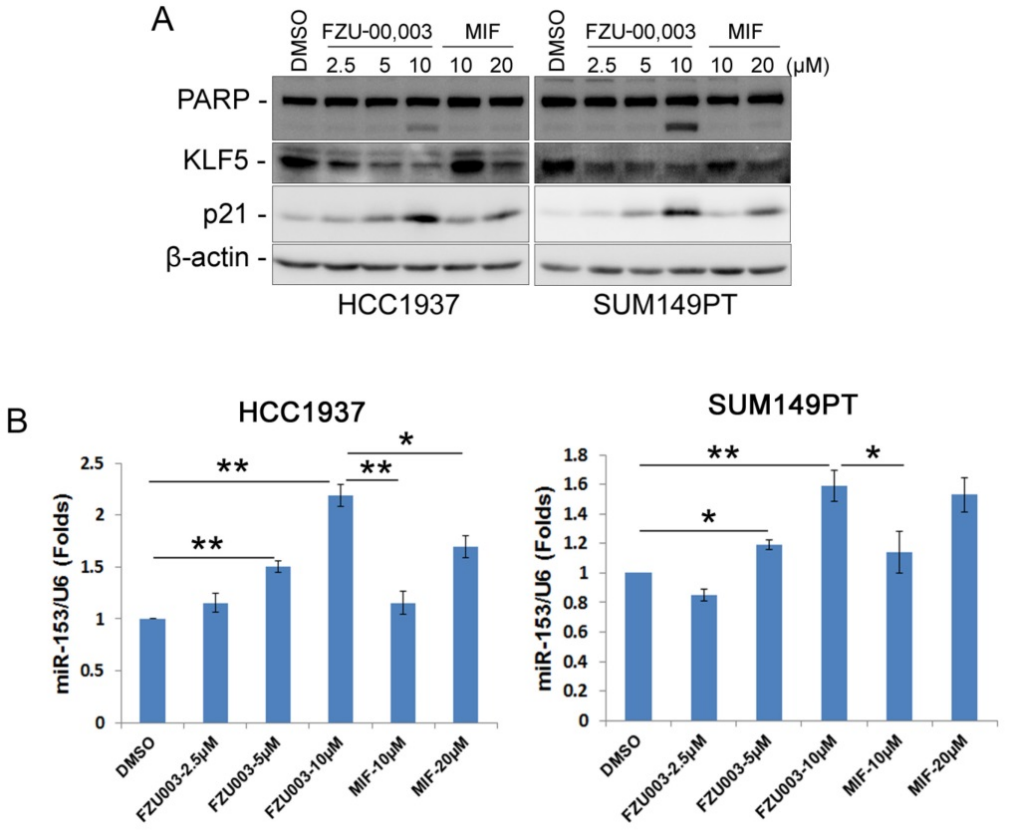
** FZU-00,003 suppressed KLF5 expression via inducing the expression of miR-153 in TNBC cells. A.** FZU-00,003 down-regulated KLF5 protein levels and induced the cleavage of PARP in a dosage-dependent manner in TNBC cells. Both HCC1937 and SUM149PT cells were treated with FZU-00,003 or MIF at indicated dosages for 24 hours. DMSO was added as the negative control. The cells were then collected for WB analysis. β-actin was used as the loading control. **B.** FZU-00,003 induced miR-153 expression more effectively than MIF did. FZU-00,003 or MIF treated cells were collected with Trizol for RNA extraction. Total RNA was then subjected to miRs reverse transcription and QPCR for detection of miR-153 expression. The expression of U6 was detected as the internal control.

As we have demonstrated previously that MIF suppressed TNBC cell growth and survival partially through down-regulating the expression of KLF5 through inducing the expression of miR-153 13, we then checked whether FZU-00,003 also suppresses KLF5 expression in TNBC cells. Indeed, FZU-00,003 suppressed KLF5 expression at much lower concentrations than MIF did in both HCC1937 and SUM149PT cell lines (Fig. 3A). Compared to MIF, FZU-00,003 induced more cell cycle dependent kinase inhibitor p21, whose transcription is well known to be suppressed by KLF5 24. Consistently, FZU-00,003 induced more apoptosis as evidenced by the induction of cleaved PARP than MIF did (Fig. 3A).

Since miR-153 is responsible for MIF to suppress KLF5 expression 13, we next investigated the effects of FZU-00,003 on miR-153 induction. As expected, FZU-00,003 induced more miR-153 at lower concentrations than MIF did (Fig. 3B).

### FZU-00,003 decreases cell viability partially via miR-153-KLF5 axis

**Figure 4:**
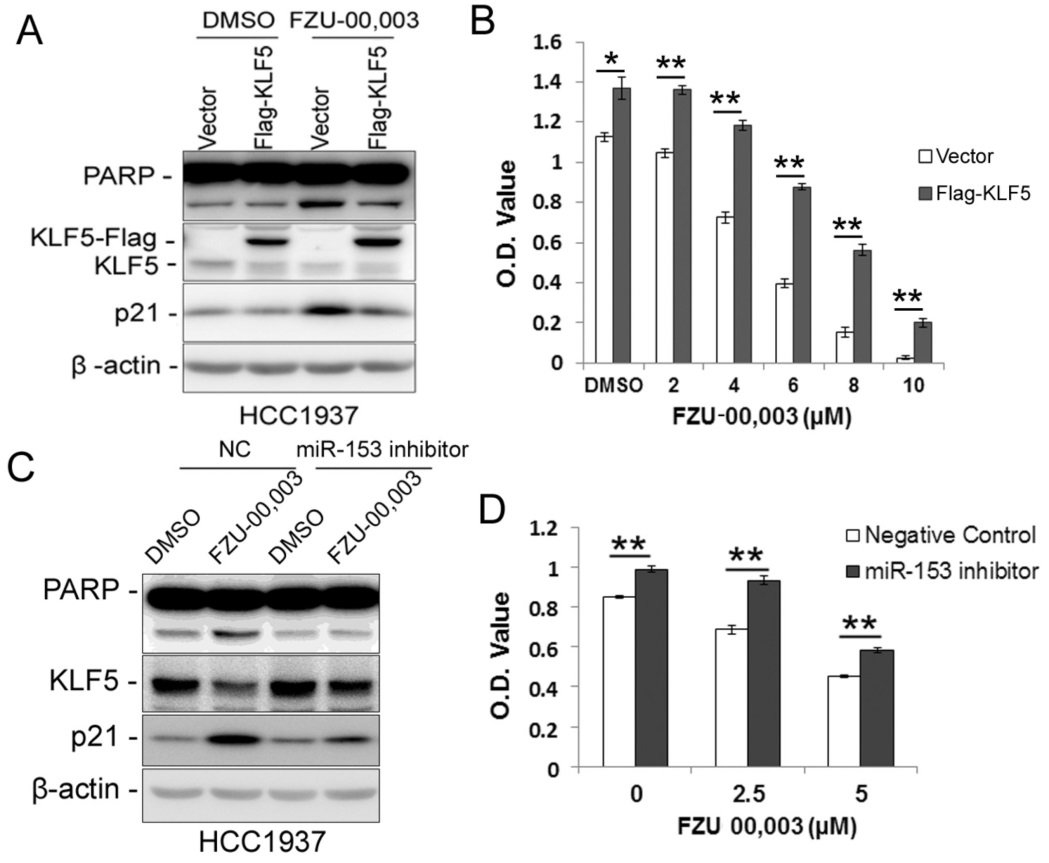
** Ectopic over-expression of KLF5 partially rescues FZU-00,003 induced apoptosis and cell viability reduction in HCC1937. A.** KLF5 over-expression decreases FZU-00,003-induced PARP cleavage in HCC1937. HCC1937 cells were infected with pCDH-Flag -KLF5 or vector control and treated with 5μM FZU-00,003 for 24 hours. The apoptosis marker cl-PARP was detected by WB. **B.** Ectopic expression of KLF5 in HCC1937 partially rescued the FZU-00,003 induced cell viability reduction.HCC1937 cell were infected with pCDH-Flag-KLF5 or vector control and treated with FZU-00,003 at indicated concentrations for 48 hours before the cells were fixed for SRB assays. **C.** miR-153 inhibitor decreases FZU-00,003-induced KLF5 suppression and PARP cleavage in HCC1937. HCC1937 cells were transfected with miR-153 inhibitor or negative control and treated with 5μM FZU-00,003 for 24 hours. **D.** miR-153 inhibitor partially rescued the FZU-00,003 induced cell viability reduction in HCC1937. HCC1937 cells were transfected with miR-153 inhibitor or negative control and treated with FZU-00,003 at indicated concentrations for 48 hours before the cells were fixed for SRB assays. *, P<0.05, **, P<0.01, t-test.

Since FZU-00,003 regulates *miR-153-KLF5 axis* and decreased cell viability in TNBC, we first tested whether FZU-00,003 decreased cell viability through down-regulating KLF5 expression. We overexpressed KLF5 in HCC1937 and treated the cells with FZU-00,003. Indeed, ectopic overexpression of KLF5 significantly reduced FZU-00,003-induced loss of cell viability and apoptosis indicated by PARP cleavage (Fig. 4A-B). Meanwhile, over-expression of KLF5 rescued the induction of p21 by FZU-00,003 (Fig. 4A). Meanwhile, we further validated whether FZU-00,003 inhibits the KLF5 expression and cell viability through inducing the miR-153. HCC1937 cells were transfected with miR-153 inhibitors followed by treating with FZU-00,003. Indeed, miR-153 inhibitors partially rescued MIF-induced KLF5 decrease, loss of cell viability and apoptosis indicated by PARP cleavage (Fig. 4C-D).

### FZU-00,003 suppresses TNBC cell growth *in vivo*

**Figure 5:**
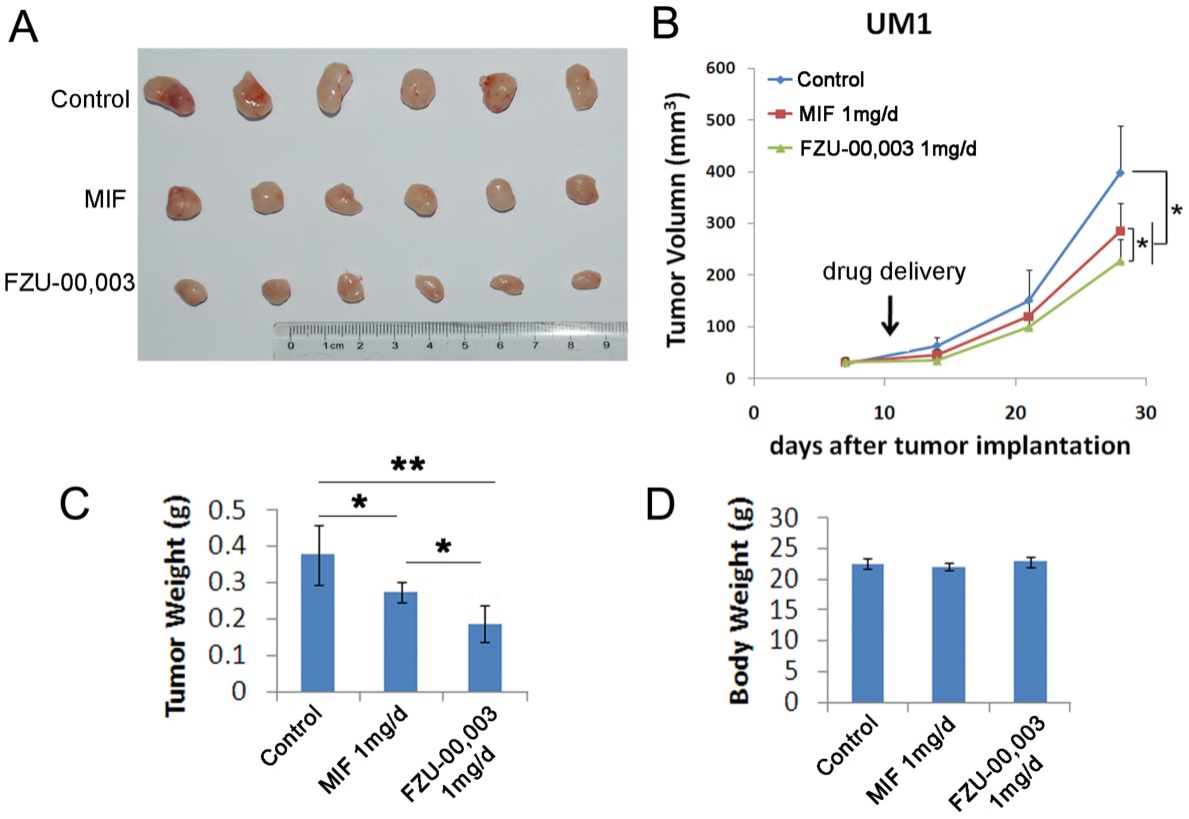
** FZU-00,003 suppresses UM1 xenograft tumor growth *in vivo*. A-B.** FZU-00,003 suppressed UM1 tumor growth in Balb/c nude mice. UM1 cells were injected into the fat pat of female Balb/c nude mice. When the average tumor size reached about 50 mm^3^ after inoculation, the mice were randomly and equally distributed into three groups (n=6/group): sesame oil control, 1 mg/d FZU-00,003 and 1 mg/d MIF. Tumor size was measured twice per week for 3 weeks. Tumors were collected 3 weeks after drug treatment. **C.** FZU-00,003 significantly decreased tumor weights compared to the MIF and control groups (*, p<0.05,**, p<0.01,t-test). **D.** Both FZU-00,003 and MIF did not affect the body weight of the mice. The mice were weighed at the end of the experiment.

FZU-00,003 suppressed TNBC cell growth and induced apoptosis *in vitro*, we then determined whether FZU-00,003 suppresses tumor growth *in vivo*. We established UM1 PDX xenografts and treated the mice with 1 mg/d FZU-00,003 or control for 3 weeks, MIF was also tested for comparison. As expected, FZU-00,003 significantly inhibited UM1 tumor growth better than MIF (Fig. 5A-C) in nude mice without affecting the body weight of the mice (Fig. 5D).

## Discussion

As the most malignant subtype of breast cancers, TNBC has the poorest prognosis but does not have effective targeted therapies. We have previously demonstrated that MIF, a synthetic PR antagonist that has been safely used as an abortifacient and an emergency contraceptive for decades 21, has an anti-tumor activity in basal TNBC cells through inhibiting KLF5 expression at relatively high doses (10-20 μM) 13. A clinical trial revealed that the peak MIF plasma concentration is about 10 μM, which is close to the effective concentration we investigated in breast cancer cells* in vitro*, 1-2 hours and remains in the micromolar range for the next 24-48 hours after a single dose of 200 mg MIF administration in health female volunteers 25. Nevertheless, a Phase II study of MIF (200 mg daily) in 28 postmenopausal patients with PR-positive breast cancer suggested that only 10.7% of patients responded to MIF alone 26. In order to improve the anticancer activity of MIF, we synthesized 17 mifepristone derivatives by modifying the methyl moiety in the sensitive metabolism region.

Remarkably, several newly synthesized mifepristone analogs have enhanced anticancer effects against two TNBC cell lines SUM149PT and HCC1937 compared to MIF. In this study, one of the novel MIF analogs FZU-00,003 was characterized in details. In comparison to MIF, FZU-00,003 (5-10 μM) exhibited more notable growth inhibitory and apoptosis-promoting effects in SUM149PT and HCC1937 TNBC cell lines. Importantly, FZU-00,003 (1 mg/d) significantly suppressed TNBC PDX xenograft tumor (UM1) growth *in vivo* without affecting mouse body weight.

Our previous studies demonstrated that KLF5 is highly expressed in basal TNBC cell lines and depletion of KLF5 significantly inhibits TNBC xenograft growth *in vivo* 19. Yagi et al delivered KLF5 siRNA into prostate cancer-bearing mice and significant suppressed PC-3 prostate tumor growth 27. Bialkowska et al. identified two small molecules suppressing the KLF5 expression and significantly inhibited colorectal cancer cell proliferation 28. More recently, our and other groups have reported that pharmacological inhibition of KLF5 by various inhibitors significantly suppressed cancer cell growth and/or survival. Curcumin suppresses bladder cancer cell growth through down-regulating KLF5 expression 29. ML264, a small molecule inhibitor of KLF5, potently inhibits proliferation of colorectal cancer cells 30. We recently reported metformin inhibits KLF5 expression and cancer stem cell in basal TNBC 14. All these data suggest that KLF5 could serve as a therapeutic target for different cancers, including breast cancer, colon cancer, prostate cancer and bladder cancer.

FZU-00,003 more efficiently down-regulated KLF5 expression through inducing miR-153 in basal TNBC cell lines compared to MIF. Moreover, both ectopic over-expression of KLF5 and miR-153 inhibitor partially rescued FZU-00,003 caused reduction of cell viability in HCC1937 indicated that FZU-00,003, at least partially, suppressed TNBC cell survival through miR-153/KLF5 axis. Of course, we could not exclude the possibility that targets other than KLF5 are involved in the anti-TNBC functions of FZU-00,003, which still need to be investigated.

Besides TNBC cells, FZU-00,003 also showed strong survival inhibition effects in other subtypes of breast cancer (Fig 1C), indicating FZU-00,003 may also be effective in treating luminal and HER2 positive breast cancers through other mechanisms since KLF5 is lowly expressed in these subtypes of breast cancer cells 18. Meanwhile, other cancers, including colon cancer, prostate cancer and bladder cancer, etc., with high KLF5 expression may also benefit from FZU-00,003 treatment. Although FZU-00,003 suppressed breast cancer cell survival at much lower dosages than MIF did, it was still used at micromole scale, implicating that further scaffold repurposing and structural optimization is still needed to obtain even more potent analogs in the future.

In conclusion, FZU-00,003 may serve as a better lead compound for the treatment of highly aggressive triple-negative breast cancers compared to MIF. Further anticancer mechanism investigation revealed that FZU-00,003 induces the expression of miR153 and inhibits KLF5 expression, like MIF but more efficiently. Preclinical studies will be needed to promote the clinical use of this compound in the future.

## Supplementary Material

Supplementary tables.
