# Supplementary material for: Mifepristone Derivative FZU-00,003 Suppresses Triple-negative Breast Cancer Cell Growth partially via miR-153-KLF5 axis
Source: Int J Biol Sci. 2020 Jan 1;16(4):611–9. doi: 10.7150/ijbs.39491 (PMC6990921; doi:10.7150/ijbs.39491)
Supplement: Supplementary file 1 — Supplementary tables. [file ijbsv16p0611s1.pdf]

Supplementary Table 1: Primer sequences used in this study

Names	Sequences (5' to 3')
U6	CGCAAGGATGACACGCAAATTC
miR-153	TTGCATAGTCACAAAAGTGATC

Supplementary Table 2: Chemical structures of 17 MIF derivatives

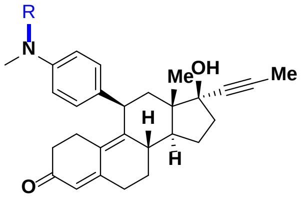

Compound	R =	Compound	R =
MIF	Me	*FZU-00,009*	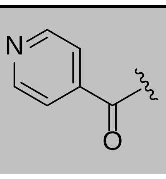
FZU-00,001	H	*FZU-00,010*	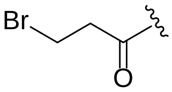
FZU-00,002	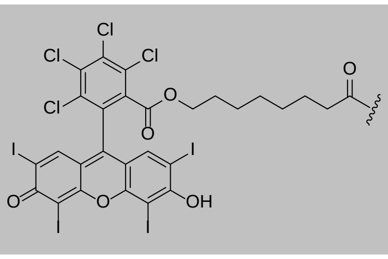	*FZU-00,011*	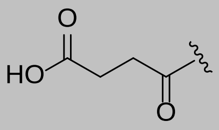
FZU-00,003	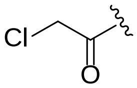	*FZU-00,012*	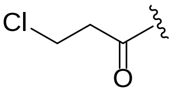
FZU-00,004	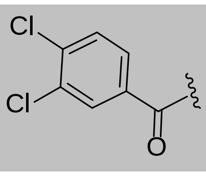	*FZU-00,013*	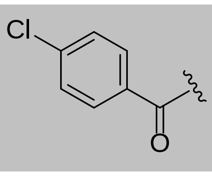
FZU-00,005	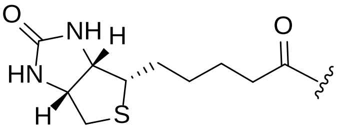	*FZU-00,014*	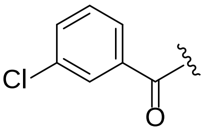
FZU-00,006	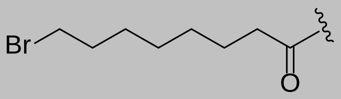	*FZU-00,015*	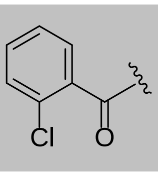
FZU-00,007	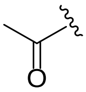	*FZU-00,016*	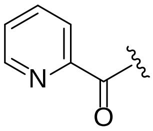
FZU-00,008	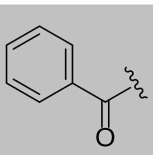	*FZU-00,017*	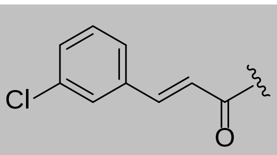
